# Closed-Loop Fuzzy Energy Regulation in Patients With Hypercortisolism via Inhibitory and Excitatory Intermittent Actuation

**DOI:** 10.3389/fnins.2021.695975

**Published:** 2021-08-09

**Authors:** Hamid Fekri Azgomi, Jin-Oh Hahn, Rose T. Faghih

**Affiliations:** ^1^Computational Medicine Lab, Department of Electrical and Computer Engineering, University of Houston, Houston, TX, United States; ^2^Department of Mechanical Engineering, University of Maryland, College Park, MD, United States

**Keywords:** closed-loop, energy state, cortisol, hypercortisolism, Bayesian estimation, wearable, fuzzy control

## Abstract

Hypercortisolism or Cushing's disease, which corresponds to the excessive levels of cortisol hormone, is associated with tiredness and fatigue during the day and disturbed sleep at night. Our goal is to employ a wearable brain machine interface architecture to regulate one's energy levels in hypercortisolism. In the present simulation study, we generate multi-day cortisol profile data for ten subjects both in healthy and disease conditions. To relate an internal hidden cognitive energy state to one's cortisol secretion patterns, we employ a state-space model. Particularly, we consider circadian upper and lower bound envelopes on cortisol levels, and timings of hypothalamic pulsatile activity underlying cortisol secretions as continuous and binary observations, respectively. To estimate the hidden cognitive energy-related state, we use Bayesian filtering. In our proposed architecture, we infer one's cognitive energy-related state using wearable devices rather than monitoring the brain activity directly and close the loop utilizing fuzzy control. To model actuation in the real-time closed-loop architecture, we simulate two types of medications that result in increasing and decreasing the energy levels in the body. Finally, we close the loop using a knowledge-based control approach. The results on ten simulated profiles verify how the proposed architecture is able to track the energy state and regulate it using hypothetical medications. In a simulation study based on experimental data, we illustrate the feasibility of designing a wearable brain machine interface architecture for energy regulation in hypercortisolism. This simulation study is a first step toward the ultimate goal of managing hypercortisolism in real-world situations.

## 1. Introduction

The cortisol hormone is the main stress hormone in an individual's body which is secreted in a pulsatile process (Azgomi and Faghih, 2019; Taghvafard et al., 2019; Wickramasuriya and Faghih, 2019b; Smyth et al., 2020). Cortisol secretion patterns, which are mainly controlled by the hypothalamus, are critical in assessing various functionalities such as regulating blood pressure and adjusting blood glucose levels. So, investigating changes in cortisol secretion would shed some light on one's internal energy state variations (Faghih, 2018; Wickramasuriya and Faghih, 2019b; Smyth et al., 2020). Adrenocorticotrophic hormone (ACTH) (i.e., a tropic hormone) causes the adrenal cortex to release cortisol in a pulsatile manner (Hakamata et al., 2017; Pednekar et al., 2019, 2020). The hypothalamus employs corticotrophin-releasing hormone (CRH) to stimulate the anterior pituitary to produce ACTH (Faghih, 2014; Faghih et al., 2014). Any irregular patterns in cortisol secretions (e.g., too much cortisol release, which is called hypercortisolism, or not providing a sufficient amount of cortisol, which is called hypocortisolism) may cause the imbalance in internal energy variations (Arnold, 2008; D'Angelo et al., 2015; Harris et al., 2015). These irregularities, which are common among the Cushing's patients who are exposed to the hypercortisolism, lead them to feel fatigue during the daytime and sleep problems at night (Stalder et al., 2016; Vance, 2017). Insufficient release of cortisol early in the morning may result in feeling fatigue during the day. On the other hand, high levels of cortisol in the evening might cause sleep disturbances at night (Dwyer et al., 2019).

While the initial treatment option for Cushing's disease is a surgery with a 78% success rate, evidence shows that the relapse happens in almost 13% of patients (Driessens et al., 2018). For the patients in whom the surgery is not successful or feasible, medical therapy is unavoidable (Pivonello et al., 2015). Due to recent advances in employing novel compounds that can regulate cortisol secretions, medical therapy has attracted more attention (Tritos and Biller, 2017). Nowadays, medical therapy is being suggested in different ways: pre-surgical treatment, post-surgical options for the patients that fail the surgical option, and the primary remedy for those in whom the surgery is not considered as an option (Pivonello et al., 2015).

The clinical observations in Cushing's syndrome patients clearly demonstrate a role for the HPA axis in the regulation of energy balance (Björntorp and Rosmond, 2000; Nieuwenhuizen and Rutters, 2008; Wickramasuriya and Faghih, 2019b). While there exist multiple factors to understand one's energy variations, there is not any specific method to directly infer internal energy state. Hence, it is not possible to present the evidence to show the correlation between energy state and cortisol variations. However, there is evidence that patients with irregular cortisol patterns experience fatigue during day time and disturbed sleep cycles at night. For example, authors in Pednekar et al. (2019, 2020) have shown that the patients with fibromyalgia syndrome, which is also associated with the irregular patterns in cortisol secretions, experience fatigue during the day and sleep disorders at night. Researchers in Crofford et al. (2004) identified lower cortisol levels in the patients with chronic fatigue syndrome. This evidence verifies the potential correlation between cortisol measurements and internal energy state.

As it is discussed, patients with Cushing's syndrome have disturbed circadian rhythm in their sleep cycles. In this regard, medications with inhibitory effects to lower the energy state and help the subjects with more balanced sleep cycles could be helpful. An example of these types of medications could be Melatonin. In the literature, it has been indicated that excessive cortisol secretions associated with Cushing's disease may lead to an irregular Melatonin rhythm (Zisapel et al., 2005; James et al., 2007). So, taking the advantages of Melatonin in improving sleep cycles, we can suggest using this medication for inhibitory effects. Although patients with hypercortisolism usually experience high levels of energy during the evening, they may suffer a lack of sufficient energy levels during the daytime (Pednekar et al., 2019, 2020). As a result, the need for medications to elevate the energy levels is unavoidable. Medications with excitatory effects to enhance energy state and prevent the subjects to feel fatigue during the daytime would be helpful in this regard. An example of these types of medications could be Methylphenidate. As patients with hypercortisolism suffer from not having enough energy levels in the daytime, medications like Methylphenidate could be suggested while implementing the proposed approach in the real world. In literature, it has been validated that taking two doses of Methylphenidate is significantly effective in relieving fatigue (Chaudhuri and Behan, 2004; Blockmans et al., 2006).

**Figure 1:**
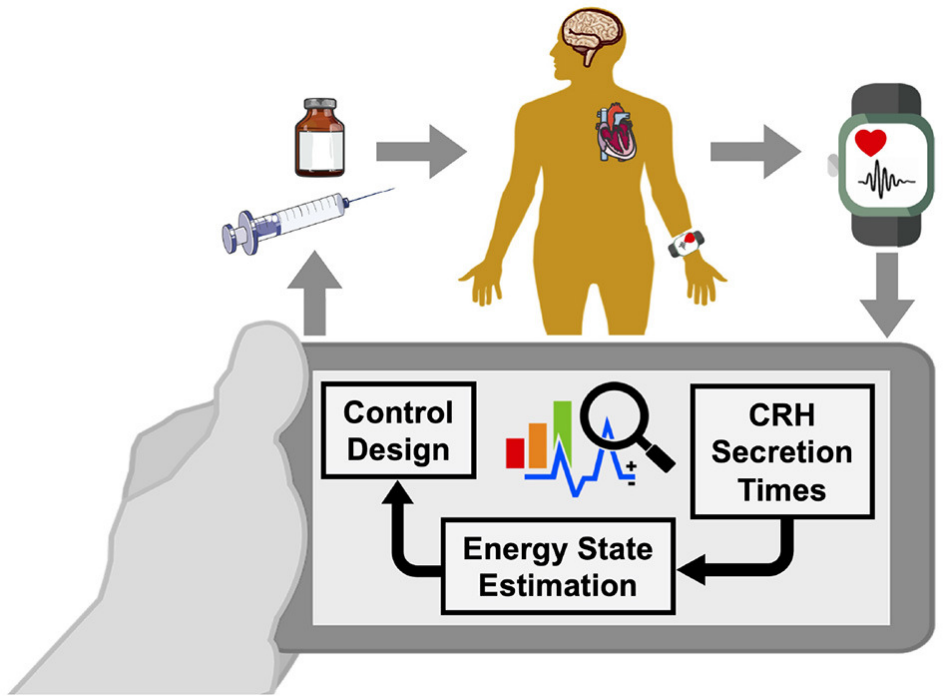
Wearable brain machine interface architecture. Blood cortisol data is being monitored by a wearable-type device. Analyzing the data collected from the human in the loop, we estimate corticotrophin-releasing hormone (CRH) secretion times that result in cortisol secretion. Then, we estimate a hidden energy state. Finally, the designed control algorithm would determine appropriate time and the dosage of medications, and will result in regulating the energy state in patients with hypercortisolism.

Due to the potential medications' side-effects, tolerance, and resistance that a person shows against the use of specific medications, it is highly important to establish a supervision layer that enables automated regulation of medication usage (Fleseriu et al., 2019). We propose our approach by taking the advantages of wearable-type devices capable of monitoring blood cortisol in a non-invasive way as a feedback modality for such supervision. The proposed approach is the first attempt to automate the regulation of medications required to manage the energy levels in patients with hypercortisolism in a closed-loop manner (Figure 1).

Recently, there has been an increased interest in employing control theory in advancing modern medication therapies such as goal-directed fluid therapy (Rinehart et al., 2011), cardiopulmonary management (Gholami et al., 2011), fluid resuscitation (Jin et al., 2019), and medically induced coma (Liberman et al., 2013; Yang and Shanechi, 2016). In a similar way, and considering how irregular cortisol secretion patterns affect energy state in patients with hypercortisolism, we leverage control theory in regulating energy variations in these patients. While there exist medications effective in managing energy levels, there is still a lack of closed-loop and automated architecture for making the decisions on the time and dosage of the medications in real-time. Hence, we construct a virtual patient environment based on the experimental cortisol data for further analysis. Then, we design the control algorithm that can determine the time and dosage of hypothetical simulated medications in a real-time automated fashion.

As someone's energy variations are influenced by changes in their cortisol levels, the objective of this research is to regulate the energy state by monitoring the cortisol secretion patterns. To model the internal energy state and relate it to the cortisol variations, we utilize the state-space model presented in Wickramasuriya and Faghih (2019b). To close the loop, we simulate hypothetical medication dynamics and develop a control system. In the present simulation study, we apply hypothetical medication dynamics as the actuation in a real-time closed-loop brain machine interface architecture (Azgomi and Faghih, 2019; Azgomi et al., 2019). As presented in Figure 1, a wearable device measures the cortisol data in a non-invasive manner. We infer the CRH secretion times via a deconvolution algorithm (Faghih, 2014; Faghih et al., 2014; Amin and Faghih, 2018, 2019a,b,c; Pednekar et al., 2019, 2020). We use the state-space approach (Brown et al., 2001; Wickramasuriya and Faghih, 2019b) to link the CRH secretion times, which cause the fluctuations in cortisol levels (Faghih et al., 2015b; Azgomi and Faghih, 2019; Wickramasuriya and Faghih, 2019b, 2020a), to the internal energy state. This state-space representation tracks the internal energy state continuously and provides the capability of utilizing the control systems theory to close the loop. To estimate the hidden cognitive energy-related state in real-time, we employ Bayesian filtering method (Wickramasuriya and Faghih, 2019b). By incorporating hypothetical dynamical system model of medications effective in both decreasing and increasing energy levels (Blockmans et al., 2006; James et al., 2007), and designing a fuzzy controller, we close the loop to regulate the energy state in patients with hypercortisolism in a simulation environment.

In section 2, we explain the steps required for creating the virtual patient environment. We also discuss the state-space model along with the real-time estimation process. We then incorporate the hypothetical medication dynamics and propose a knowledge-based control system to close the loop in real-time. In section 3, we present the outcome of implementing the proposed approach in regulating the energy state in patients with hypercortisolism. More particularly, we present the results on two classes of patients: (1) who do not have the circadian rhythm in their cortisol profiles, and (2) who have the circadian rhythm in their cortisol profiles. The final results demonstrate that our proposed real-time architecture can not only track one's energy state, but also regulate the energy variations in patients with hypercortisolism utilizing the simulated medication dynamics. Section 4 points out the implications of our findings. This simulation study based on the experimental data is the first step toward treating other hormone-related disorders.

## 2. Methods

**Figure 2:**
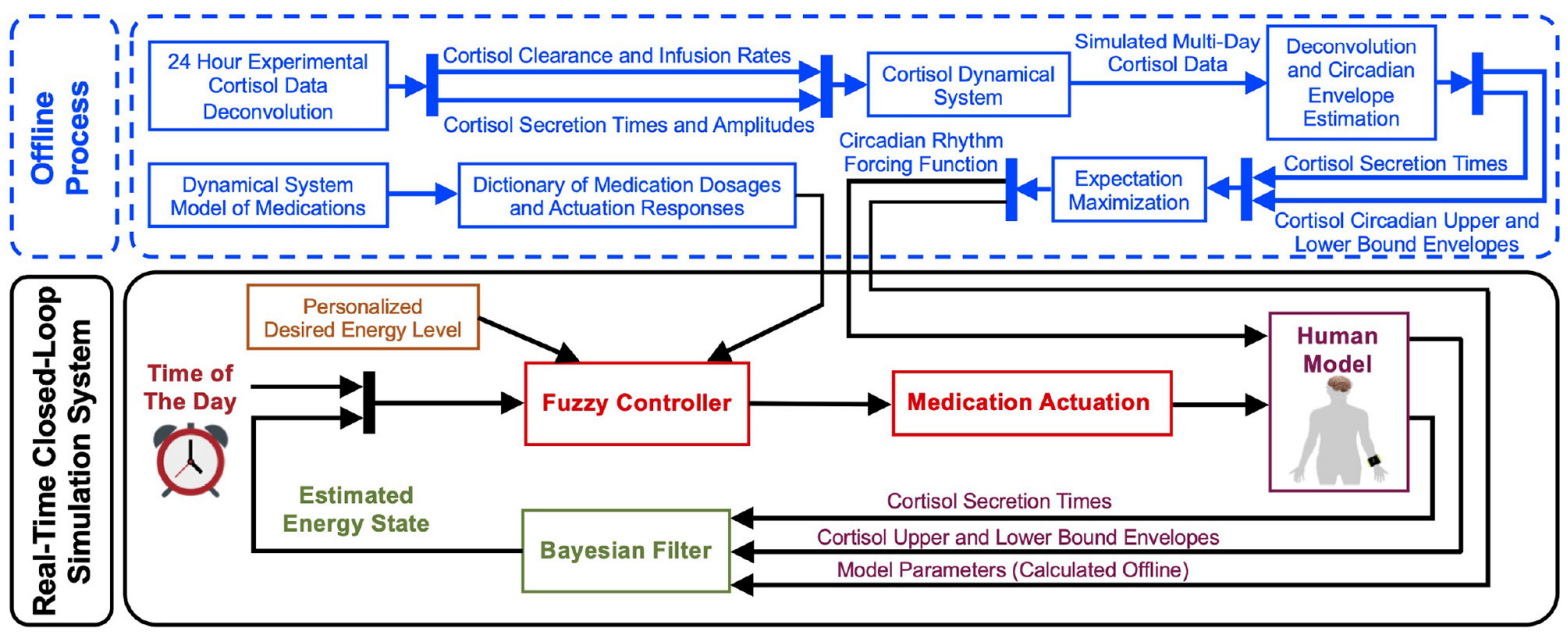
Real-time closed-loop energy regulation. The offline process includes extending the cortisol data profiles for multi-day data creation, simulating the medication dynamics, and estimating the filter parameters required for the closed-loop section. In the real-time closed-loop system, using the state-space representation, the internal energy state gets related to the brain secretion times that result in the cortisol secretions. Then, a Bayesian decoder employs the brain secretion times and the upper and lower cortisol envelopes as the observations to estimate the energy state. Finally, employing the personalized desired energy levels and information regarding the actuation dynamics, a fuzzy controller is generated. The closed-loop system automates the time and dosage of applying simulated medications to regulate the energy state.

Figure 2 illustrates an overview of the proposed closed-loop architecture. The present study consists of two main parts: the offline process and the real-time closed-loop simulation environment. In the offline part, we first generate multi-day cortisol data for multiple subjects based on their experimental data collected over 24 h. Although there are recent advances in monitoring cortisol levels using wearable devices (Venugopal et al., 2011; Parlak et al., 2018; Parlak, 2021), there is still a lack of technologies for real-time multi-day cortisol data collection. Hence, to design a virtual patient environment, we first follow the results from Wickramasuriya and Faghih (2019b), Brown et al. (2001), and Lee et al. (2016) to simulate cortisol profiles in both healthy subjects and Cushing's patients. To extend our preliminary results presented in Azgomi and Faghih (2019), we simulate data for ten subjects (Faghih, 2014). This offline process enables us to examine the performance of the proposed architecture in multiple cases. By performing deconvolution algorithm, we infer the cortisol secretion times and the circadian upper and lower envelopes. Utilizing Expectation Maximization (EM) approach, we estimate the circadian rhythm forcing function along with model parameters. In the offline stage, we also model dynamical systems for hypothetical medications with both inhibitory (i.e., medications to lower the energy levels) and excitatory (i.e., medications to elevate the energy levels) effects.

As depicted in the bottom section of Figure 2, we take the circadian rhythm forcing function in the real-time simulation system and relate the internal energy state to the cortisol secretion times and cortisol upper and lower bound envelopes using the state-space approach. Employing the Bayesian filtering, which uses the estimated model parameters calculated with the offline EM algorithm, we estimate the hidden energy-related state in real-time. Incorporating the dynamical system model of medications and the personalized desired levels of energy, we design a fuzzy controller to close the loop. The deigned control system will take the energy state estimate and determine the time and dosage of each medication as the actuation in the loop. Hence, it controls cortisol variations which will result in energy regulation.

### 2.1. Data Simulation

Due to the lack of multi-day experimental measurements of healthy subjects and the patients with Cushing's disease, we first simulate multi-day cortisol data profiles (Brown et al., 2001; Faghih, 2014; Lee et al., 2016; Wickramasuriya and Faghih, 2019b). Following Faghih et al. (2014) and Brown et al. (2001), cortisol secretion process could be assumed to follow a second-order stochastic differential equation:

(1) $$\begin{array}{l} \frac{dCort_{1}\left(t\right)}{dt}=-\zeta_{1}Cort_{1}\left(t\right)+n\left(t\right), \end{array}$$

(2) $$\begin{array}{l} \frac{dCort_{2}\left(t\right)}{dt}=\zeta_{1}Cort_{1}\left(t\right)-\zeta_{2}Cort_{2}\left(t\right), \end{array}$$

where *Cort*_1_(*t*) and *Cort*_2_(*t*) are cortisol concentration in adrenal glands and plasma space at time *t*, respectively (Faghih, 2014). Moreover, ζ_1_ stands for cortisol infusion rate from adrenal gland to the blood, ζ_2_ corresponds to the cortisol clearance rate by the liver (Brown et al., 2001; Faghih et al., 2014). In addition, *n*(*t*) represents secretory events (pulses) underlying cortisol release. The output equation *y*_*k*_ = *Cort*_2_(*k*) + ψ_*k*_, where *Cort*_2_(*k*) is the discretized cortisol concentration in plasma with $\psi_{k}\sim N\left(0,\sigma_{\psi }^{2}\right)$ as the measurement noise with variance $\sigma_{\psi }^{2}$. We employ estimated model parameters ζ_1_ and ζ_2_ derived in Faghih et al. (2014). The details of this information are presented in Supplementary Material.

To model cortisol secretory events *n*(*t*), we follow the approach presented in Brown et al. (2001).

- Healthy Profiles: We use the gamma distribution for pulse inter-arrival times and Gaussian distribution for pulse amplitudes (Brown et al., 2001). The corresponding parameters for gamma distribution are α = 54 and β = 39. The pulse amplitude follows a Gaussian distribution $H_{k}\sim N\left(\mu_{k},k_{k}^{2}\right)$, where $\mu_{k}=6.1+3.93\sin\left(\frac{2\pi k}{1440}\right)-4.75\cos\left(\frac{2\pi k}{1440}\right)-2.53\sin\left(\frac{4\pi k}{1440}\right)-3.76\cos\left(\frac{4\pi k}{1440}\right)$ and $k_{k}=0.1\sqrt{\mu_{k}}$ (Azgomi and Faghih, 2019; Wickramasuriya and Faghih, 2019b).

To simulate the data for patients with Cushing's disease, we consider two cases: (1) Cushing's patients without circadian rhythms in their cortisol profiles, and (2) Cushing's patients with circadian rhythms in their cortisol profiles. While cortisol variations in patients with Cushing's disease do not follow normal circadian rhythms, at the very early stages of the disease, the circadian rhythms might be slightly dysregulated (Van den Berg et al., 1995; Wickramasuriya and Faghih, 2019b).

- Cushing's patients without circadian rhythm: We follow (Van den Berg et al., 1995; Lee et al., 2016) and consider the inter-arrival times following a gamma distribution that belong to the range of 59 ± 11 min. Regarding the pulse amplitudes, we assume they are within the range of 38±2.5 $\mu \text{gd}{\text{L}}^{-1}\overset{-1}{\min}$, following a Gaussian distribution (Azgomi and Faghih, 2019; Wickramasuriya and Faghih, 2019b),
- Cushing's patients with circadian rhythm: We employ $\mu_{k}=38.5+1.93\sin\left(\frac{2\pi k}{1440}\right)-1.6\cos\left(\frac{2\pi k}{1440}\right)-1.5\sin\left(\frac{4\pi k}{1440}\right)-3.5\cos\left(\frac{4\pi k}{1440}\right)$, $k_{k}=\frac{2.5}{\sqrt{38\mu_{k}}}$ as the Gaussian distribution parameters in the pulse amplitudes and the same gamma inter-arrival time distribution as described previously for the Cushing's patients with circadian rhythm.

**Figure 3:**
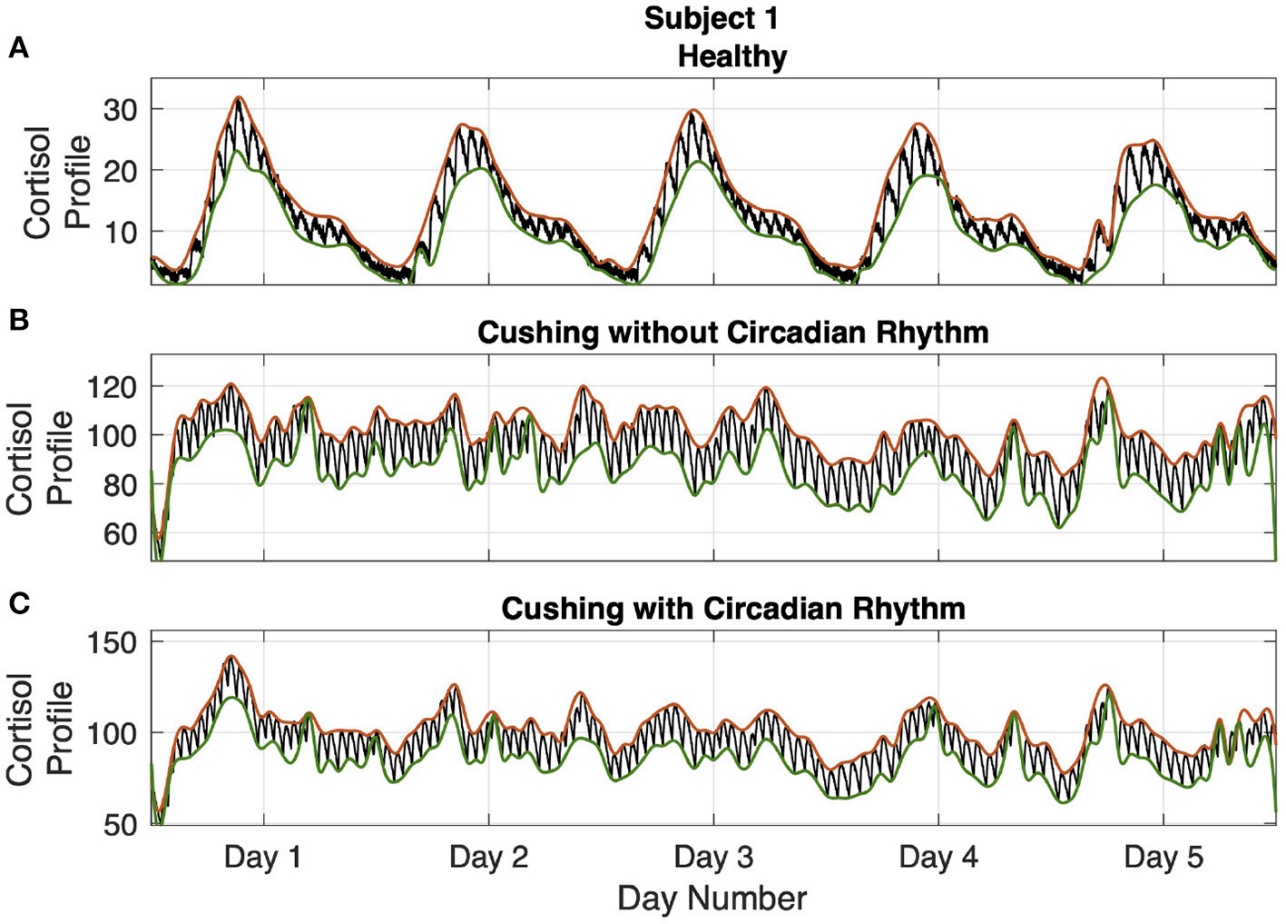
Simulated multi-day cortisol profile - Subject 1. Panel **(A)** displays the healthy profile, panel **(B)** shows the profile associated with the Cushing's patients without circadian rhythm, and panel **(C)** depicts the profiles associated with the Cushing's patients with circadian rhythm. Each panel displays cortisol levels (black curve), upper bound envelopes (orange curve), and lower bound envelopes (green curve).

Employing the model parameters ζ_1_ and ζ_2_ provided in Supplementary Material and a vector input of pulse timings and amplitudes *n*(*t*) presented above, we simulate the cortisol profiles. We employ coupled differential equations (1) and (2), and add measurement noise to generate cortisol profile data for different subjects in three different situations. More particularly, we simulate the cortisol profiles associated with healthy subjects, Cushing's patients with circadian rhythm in their cortisol profiles and Cushing's patients without circadian rhythm in their cortisol profiles over 5 days for further analysis (Brown et al., 2001; Faghih, 2014, 2018). The resulting multi-day cortisol profiles are presented in Supplementary Material. As an example, the results associated with subject 1 are depicted in Figure 3.

### 2.2. State-Space Modeling

Cortisol dynamical system explained above will generate the cortisol observations for our virtual patient environment. We employ the state-space approach presented in Azgomi and Faghih (2019); Wickramasuriya and Faghih (2019b) to relate the hidden cognitive energy-related state to cortisol variations. The state-space approach lets us systematically track internal energy state and control it in real-time (Smith et al., 2004). We model the cognitive energy-related state as the following first-order state-space representation:

(3) $$\begin{array}{l} x_{k}=\rho x_{k-1}+u_{k}+\epsilon_{k}+I_{k}, \end{array}$$

where *x*_*k*_ is the hidden internal energy-related state, ρ is a person-specific parameter, *u*_*k*_ is the control input, $\epsilon_{k}\sim N\left(0,\sigma_{\epsilon }^{2}\right)$ is the process noise, and *I*_*k*_ is being considered as the forcing function that keeps the energy variations during wakefulness and sleep in 24 h periods. By analyzing the simulated cortisol profiles (Wickramasuriya and Faghih, 2019b), we design the following harmonic forcing function:

(4) $$\begin{array}{l} I_{k}=\overset{2}{\underset{i=1}{\sum }}\alpha_{i}\sin\left(\frac{2\pi ik}{1440}\right)+\beta_{i}\cos\left(\frac{2\pi ik}{1440}\right), \end{array}$$

where the coefficients α_*i*_ and β_*i*_ along with parameter ρ in (3) for each subject/case are derived using the EM algorithm explained in Wickramasuriya and Faghih (2019b). These parameters are presented in Supplementary Material.

Analyzing the discretized cortisol data at a 1 min time resolution, we observe that the presence or absence of the cortisol pulses builds a binary point process (Wickramasuriya and Faghih, 2019b). Hence, we assume the probability of receiving pulses associated with CRH secretion times that results in cortisol secretion, *c*_*k*_, follows a Bernoulli distribution:

(5) $$\begin{array}{l} P\left(c_{k}|p_{k}\right)=p_{k}^{c_{k}}{\left(1-p_{k}\right)}^{1-c_{k}}, \end{array}$$

where the probability *p*_*k*_ is connected to the energy state *x*_*k*_ by the following sigmoid function:

(6) $$\begin{array}{l} p_{k}=\frac{1}{1+e^{-\left(\gamma_{0}+\gamma_{1}x_{k}\right)}}. \end{array}$$

This model relates the probability *p*_*k*_ of observing a CRH pulse event *c*_*k*_ to the energy state *x*_*k*_ through person-specific baseline parameters γ_0_ and γ_1_ calculated by the offline EM.

In addition to the cortisol secretion times as binary observations, we use the upper and the lower bound envelopes of the blood cortisol measurements as continuous observations to estimate the energy state *x*_*k*_ (Wickramasuriya and Faghih, 2019b). We label these two upper and lower envelopes as *R*_*k*_ and *S*_*k*_, respectively. Assuming there exists a linear relationship between these envelopes and the corresponding state *x*_*k*_:

(7) $$\begin{array}{l} R_{k}=r_{0}+r_{1}x_{k}+v_{k}, \end{array}$$

(8) $$\begin{array}{l} S_{k}=s_{0}+s_{1}x_{k}+w_{k}, \end{array}$$

where $v_{k}\sim N\left(0,\sigma_{v}^{2}\right),w_{k}\sim N\left(0,\sigma_{w}^{2}\right)$, and *r*_0_, *r*_1_, *s*_0_, *s*_1_ are regression coefficients obtained by offline EM algorithm (Prerau et al., 2009; Coleman et al., 2011; Wickramasuriya and Faghih, 2019a).

It is worth mentioning that while there exist recent advances in performing deconvolution methods, there is still lack of real-time deconvolution algorithm. With real-time deconvolution tool, we directly infer the cortisol impulses *n*(*t*) in (1) and employ it in further analysis.

### 2.3. Energy State Estimation

We employ two continuous and one binary observations in the estimation process (Prerau et al., 2009; Coleman et al., 2011; Wickramasuriya and Faghih, 2019a). Taking the CRH pulse events *c*_*k*_ and the upper and lower envelopes *R*_*k*_ and *S*_*k*_ as observations, we perform Bayesian filtering (Coleman et al., 2011; Wickramasuriya and Faghih, 2020b) to estimate the hidden cognitive energy-related state mean *x*_*k*_ and its variance σ_*k*_ in two prediction and update steps:

- Prediction step:
  (9) $$\begin{array}{l} x_{k|k-1}=\rho x_{k-1|k-1}+I_{k}+u_{k}, \end{array}$$
  (10) $$\begin{array}{l} \sigma_{k|k-1}^{2}=\rho^{2}\sigma_{k-1|k-1}^{2}+\sigma_{\epsilon }^{2}. \end{array}$$
- Update step:
  (11) $$\begin{array}{l} A_{k}=\frac{\sigma_{k|k-1}^{2}}{\sigma_{v}^{2}\sigma_{w}^{2}+\sigma_{k|k-1}^{2}\left(r_{1}^{2}\sigma_{w}^{2}+s_{1}^{2}\sigma_{v}^{2}\right)}, \end{array}$$
  (12) $$\begin{array}{l} \begin{array}{c} {\hat{x}}_{k}=x_{k|k}=x_{k|k-1}+A_{k}(\gamma_{1}\sigma_{v}^{2}\left(c_{k}-p_{k}\right)\\ \;+r_{1}^{2}\sigma_{w}^{2}\left(R_{k}-r_{0}-r_{1}x_{k|k-1}\right)\\ \;+s_{1}^{2}\sigma_{v}^{2}\left(S_{k}-s_{0}-s_{1}x_{k|k-1}\right)), \end{array} \end{array}$$
  (13) $$\begin{array}{l} {\hat{\sigma }}_{k}^{2}=\sigma_{k|k}^{2}=(\frac{1}{\sigma_{k|k-1}^{2}}+\gamma_{1}^{2}p_{k}\left(1-p_{k}\right)+\frac{r_{1}^{2}}{\sigma_{v}^{2}}+\frac{s_{1}^{2}}{\sigma_{w}^{2}}{)}^{-1}. \end{array}$$

The *p*_*k*_ presented in (12) is related to the ${\hat{x}}_{k}$ by (6). Consequently, the ${\hat{x}}_{k}$ is present on both sides of (12) and we employ Newton's method to solve the update equations.

### 2.4. Dynamic System Model of Medications

The next step in closing the loop and regulating energy-related state is to model the dynamical system of hypothetical medications and include them in control design process. In this research, we focus on the medications that can lead the subjects to reach their desired energy levels (Blockmans et al., 2006; James et al., 2007). In this regard, we consider two classes of medications: (1) for elevating the energy levels required for daily activity (i.e., excitation effect), and (2) for helping the subjects to lower their energy levels in the evening which may help them experience well-ordered sleep cycles at nights (i.e., inhibition effect). To analyze how a specific medication affects one's energy levels and incorporate them in the control design process, we model their dynamics by a second-order state-space representation:

(14) $$\begin{array}{ll} \left[\begin{array}{c} {ż}_{1}\left(t\right)\\ {ż}_{2}\left(t\right) \end{array}\right] & =\left[\begin{array}{cc} -\theta_{i1} & 0\\ \theta_{i1} & -\theta_{i2} \end{array}\right]\left[\begin{array}{c} z_{1}\left(t\right)\\ z_{2}\left(t\right) \end{array}\right]+\left[\begin{array}{c} \eta \\ 0 \end{array}\right]q\left(t\right), \end{array}$$

where *i* = 1, 2 denotes the type of medications. *y*(*t*) = *z*_2_(*t*) is the estimated energy level and θ_*i*1_, θ_*i*2_ correspond the infusion rate and the clearance rate of each corresponding medication *i*, respectively. We assume ***θ***_*i*_ = [θ_*i*1_ θ_*i*2_]. In the state-space representation (14), $q\left(t\right)=q_{i}^{*}\delta \left(t-\tau_{i}^{*}\right)$ is the actuation input impulses where parameters $\tau_{i}^{*}$ and $q_{i}^{*}$ stand for time and dosage of the corresponding medication *i* (Faghih, 2014; Faghih et al., 2015a). The η term also determines if the actuation should be excitation (i.e., η = + 1 for elevating the energy level) or inhibition (i.e., η = −1 for lowering the energy level). With this representation, we analyze how using a specific dosage $q_{i}^{*}$ of medication *i* at time $\tau_{i}^{*}$ will affect the internal energy levels *z*_2_(*t*) dynamically. Solving the state-space equation (14) and considering the output equation *y*(*t*) = *z*_2_(*t*), we compute the output at each time step *j* as:

(15) $$\begin{array}{l} y_{j}=a_{j}y_{0}+{\text{b}}_{\text{j}}\text{q}+e_{j}. \end{array}$$

where $a_{j}=e^{-\theta_{i2}j}$ and ${\text{b}}_{\text{j}}=\frac{\theta_{i1}}{\theta_{i1}-\theta_{i2}}[\left(e^{-\theta_{i2}j}-e^{-\theta_{i1}j}\right)\;\left(e^{-\theta_{i2}\left(j-1\right)}-e^{-\theta_{i1}\left(j-1\right)}\right)\;\cdots \;\left(e^{-\theta_{i2}}-e^{-\theta_{i1}}\right)\;\underset{N-j}{\underset{︸}{0\;\cdots \;0}}{]}^{\prime }$. The vector input **q** consists of one non-zero element (i.e., **q** = [*q*_1_ ⋯ *q*_*N*_], where *q*_*j*_ = 0, ∀*j* except the one element $q_{i}^{*}$ at time $\tau_{i}^{*}$) and error term $e_{j}\sim N\left(0,\sigma_{e}^{2}\right)$. Forming the output for the whole time horizon *N*, we generate the vector representation **y** as the observation:

(16) $$\begin{array}{l} \text{y}={\text{A}}_{\theta }y_{0}+{\text{B}}_{\theta }\text{q}+\text{e}, \end{array}$$

where $\text{y}={\left[\begin{array}{cccc} y_{1} & y_{2} & \cdots & y_{N} \end{array}\right]}^{\prime }$, ${\text{A}}_{\theta }={\left[\begin{array}{cccc} a_{1} & a_{2} & \cdots & a_{N} \end{array}\right]}^{\prime }$, ${\text{B}}_{\theta }={\left[\begin{array}{cccc} {\text{b}}_{1} & {\text{b}}_{2} & \cdots & {\text{b}}_{\text{N}} \end{array}\right]}^{\prime }$, and $\text{e}={\left[\begin{array}{cccc} e_{1} & e_{2} & \cdots & e_{N} \end{array}\right]}^{\prime }$. To complete the system identification task, we impose the constraint ||**q**||_0_ = 1 in the corresponding parameter estimation problem (Dahleh et al., 2004). To find the optimum parameters, we solve the following optimization problem to optimize the error term *J* = **e**′**e**:

(17) $$\begin{array}{l} \underset{\begin{array}{c} \theta_{i},\text{q}\\ ||\text{q}|{|}_{0}=1 \end{array}}{\min}J=\frac{1}{2}||\text{y}-{\text{A}}_{\theta }y_{0}-{\text{B}}_{\theta }\text{q}|{|}_{2}^{2}. \end{array}$$

Given **y**, we can estimate **A**_θ_, **B**_θ_ (i.e., include ***θ***_*i*_), and **q** to obtain the actuation dynamics (Faghih et al., 2015b). As a result of this process, we simulate the way that a specific medication affects the energy levels. In the following part, we explain the control approach and close the loop.

In this *in silico* study, incorporating the hypothetical medication dynamics (14), we design the control strategy to determine the time and the dosage of each medication to regulate the estimated energy state. In the practical case, this system identification step is recommended to be performed in parallel to update the dynamical model parameters in real-time.

### 2.5. Fuzzy Control System

Fuzzy control, which is known as a knowledge-based control approach, employs the insights about the system, performs the corresponding inference, and makes the control decisions (Garibaldi and Ozen, 2007; Mendes et al., 2019; Yu et al., 2020). As an intelligent approach, it is a powerful bridge from the expertise inference to the real world (Lin et al., 2018; Azgomi et al., 2019). Any fuzzy controller includes four main parts: rule base, fuzzifier, inference engine, and defuzzifier. In the rule base, we define the rules to achieve our control objective (Zoukit et al., 2019). These IF-THEN rules are derived employing expert knowledge of the system and the corresponding constraints.

In the present study, the estimated cognitive energy-related state and the time of the day are the inputs of the fuzzy controller, and the control output is the time and dosage of the required simulated medications (Azgomi and Faghih, 2019). To design the fuzzy system, we employ information about the personalized levels of energy state and the dictionary of medication dosages and actuation responses (Figure 2). We also use two classes of actuation: exciting medications which increase the energy levels, and inhibiting medications which lower the estimated energy levels. The purpose of applying medications with exciting and inhibiting effects is to provide the required energy for daily activity (Pednekar et al., 2020) and lowering the energy-related state to result in a better sleep cycle at nights (Feelders et al., 2019), respectively. Based on the literature and nature of the medications (Blockmans et al., 2006; James et al., 2007), we consider the constraint of applying maximum two medications (i.e., control inputs) per day: one in the morning which increases energy levels, and one in the evening to lower the energy levels. The rule base of the proposed fuzzy controller is presented in Table 1.

**Table 1:** Fuzzy rule base.

**Rule #**	**Time *(Input 1)***	**Energy State *(Input 2)***	**Actuation *(Output)***
1	Early morning	High	Positive small
2	Early morning	Low	Positive big
3	Early morning	Medium	Positive medium
4	Late morning	High	Zero
5	Late morning	Low	Positive medium
6	Late morning	Medium	Positive small
7	Early evening	High	Negative medium
8	Early evening	Low	Zero
9	Early evening	Medium	Negative small
10	Late evening	High	Negative big
11	Late evening	Low	Negative small
12	Late evening	Medium	Negative medium

As an example, to clarify the structure of rules presented in Table 1, rule number 1 denotes:

- If the estimated energy state is *High*, and the time is *early in the morning* then the actuation is *positive small*.

**Figure 4:**
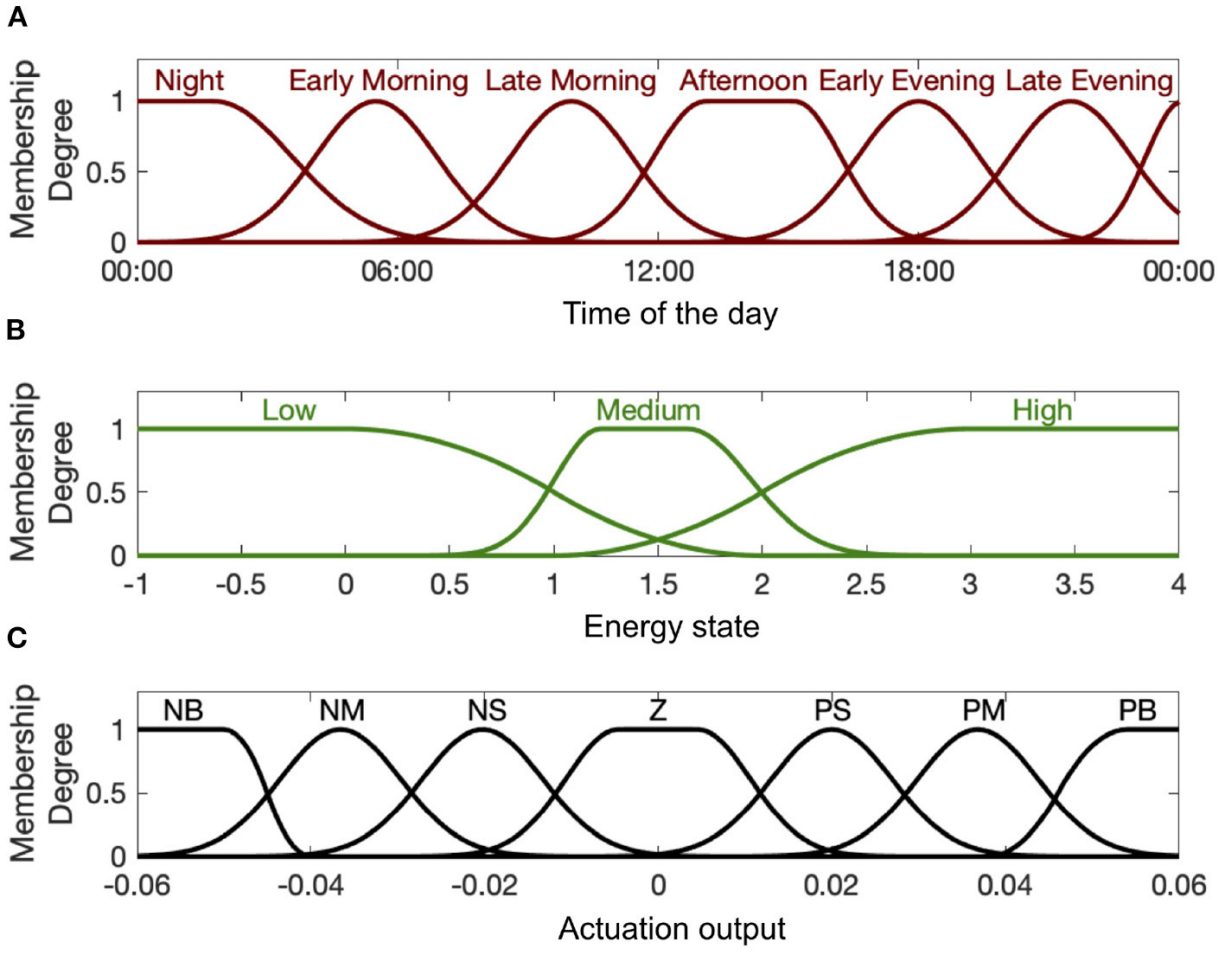
Input and output membership functions. Panel **(A)** shows the first input membership functions describing time of the day. Panel **(B)** shows the input membership functions associated with the estimated energy-related state. Panel **(C)** shows the membership functions for the actuation output (i.e., control signal *u*_*k*_). The abbreviations P, N, Z, S, M, and B stand for “Positive,” “Negative,” “Zero,” “Small,” “Medium,” and “Big,” respectively.

To quantify the linguistic variables presented in the rule base, we employ membership functions as the fuzzifiers (Azgomi et al., 2013). Investigating the simulated environment including estimated energy state, hypothetical medication dynamics, personalized levels of energy state, and the rule base, we utilize the appropriate number of relevant membership functions presented in Figure 4. As observed in Figure 4, we employ six membership functions for time of the day (input 1), three membership functions for estimated energy values (input 2), and seven membership functions for the control output to cover all cases in the rule base (Table 1).

We use *Mamdani* inference engine to execute the inference and produce fuzzy outputs (Zulfikar et al., 2018). We employ *minimum* method for both *AND* operation in the fuzzy inputs and implication process for fuzzy output generation. We also use *Maximum* method for rule output aggregation. Consequently, the final fuzzy output will be resulted as:

(18) $$\begin{array}{l} \begin{array}{c} \mu_{mamdani}\left(q\right)=\mu_{m}\left(q\right)=\underset{\begin{array}{c} j \end{array}}{\max}\left[\mu_{j}\left(q\right)\right]\\ \;=\underset{\begin{array}{c} j \end{array}}{\max}\left[min(min\left(\mu_{time}\left(t\right),\mu_{state}\left(x\right)\right),\mu_{actuation}\left(c\right))\right]\\ \;=\underset{\begin{array}{c} j \end{array}}{\max}\left[min\left(\mu_{time}\left(t\right),\mu_{state}\left(x\right),\mu_{actuation}\left(c\right)\right)\right]. \end{array} \end{array}$$

where *j* denotes the effective rules at each time step and μ_*j*_(*q*) is the resulted fuzzy set. μ_*time*_(*t*), μ_*state*_(*x*), and μ_*actuation*_(*c*) also stand for the membership functions presented in Figure 4. To demonstrate the way that this inference engine works, we explain the proposed fuzzy system (18). At each time step, the fuzzy system monitors all the rules presented in Table 1 and finds the effective rules according to the input membership functions (Figure 4). By extracting the corresponding membership degree and executing *AND* operation in each applied rule, it then performs implication between the resulted input fuzzy sets (time and the estimated energy state) and the corresponding output fuzzy membership function (medication actuation). By aggregating results from all applied rules, it generates the final fuzzy output. To produce crisp output out of the generated fuzzy outputs and applying it into the system in real-time, we employ *centroid* defuzzification method:

(19) $$\begin{array}{l} q^{*}=\frac{\int \mu_{m}\left(q\right).qdq}{\int \mu_{m}\left(q\right)dq}. \end{array}$$

At any time step where either the rules with *Zero* actuation output (Table 1) are effective, or the output q^*^ in (19) equals zero, the fuzzy system would determine no need for applied control. At the time *t* that the fuzzy system results a non-zero output (q^*^ ≠ 0), time of actuation would be derived (τ^*^ = *t*). Considering the resulted crisp output and constraint to apply maximum two medications per day, the designed control will determine the time and the amplitudes of each medication. Hence, by taking the decisions about the dosage and the desired time of the hypothetical medications [i.e., q^*^ and τ^*^ in (14)], the resulted control signal [i.e., *u*_*k*_ in (3)] will be applied to regulate the internal energy state.

## 3. Results

**Figure 5:**
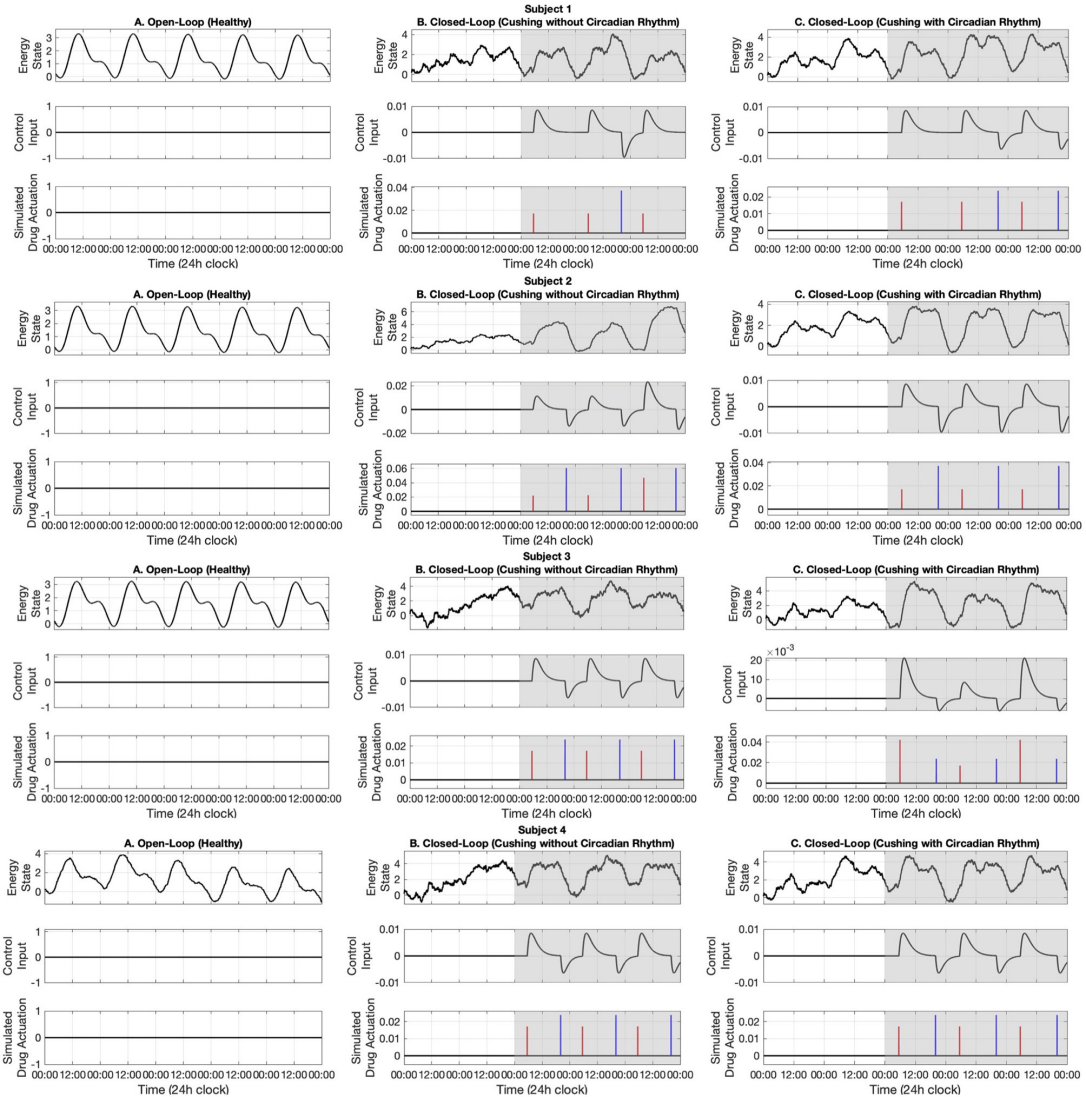
Simulated energy regulation results (Subjects 1–4). For each subject, panel **(A)** displays the open-loop results. Panel **(B)** shows closed-loop results for the Cushing's patients without circadian rhythm, while panel **(C)** shows closed-loop results for the Cushing's patients with circadian rhythm. In each panel: the top sub-panel shows the estimated cognitive energy-related state, the middle sub-panel displays the control input, and the bottom sub-panel depicts the medication injections. Red pulses are related to excitation and the blue pulses are related to inhibition. The white background indicates open-loop simulation (i.e., *u* = 0), while the gray background depicts the closed-loop results.

**Figure 6:**
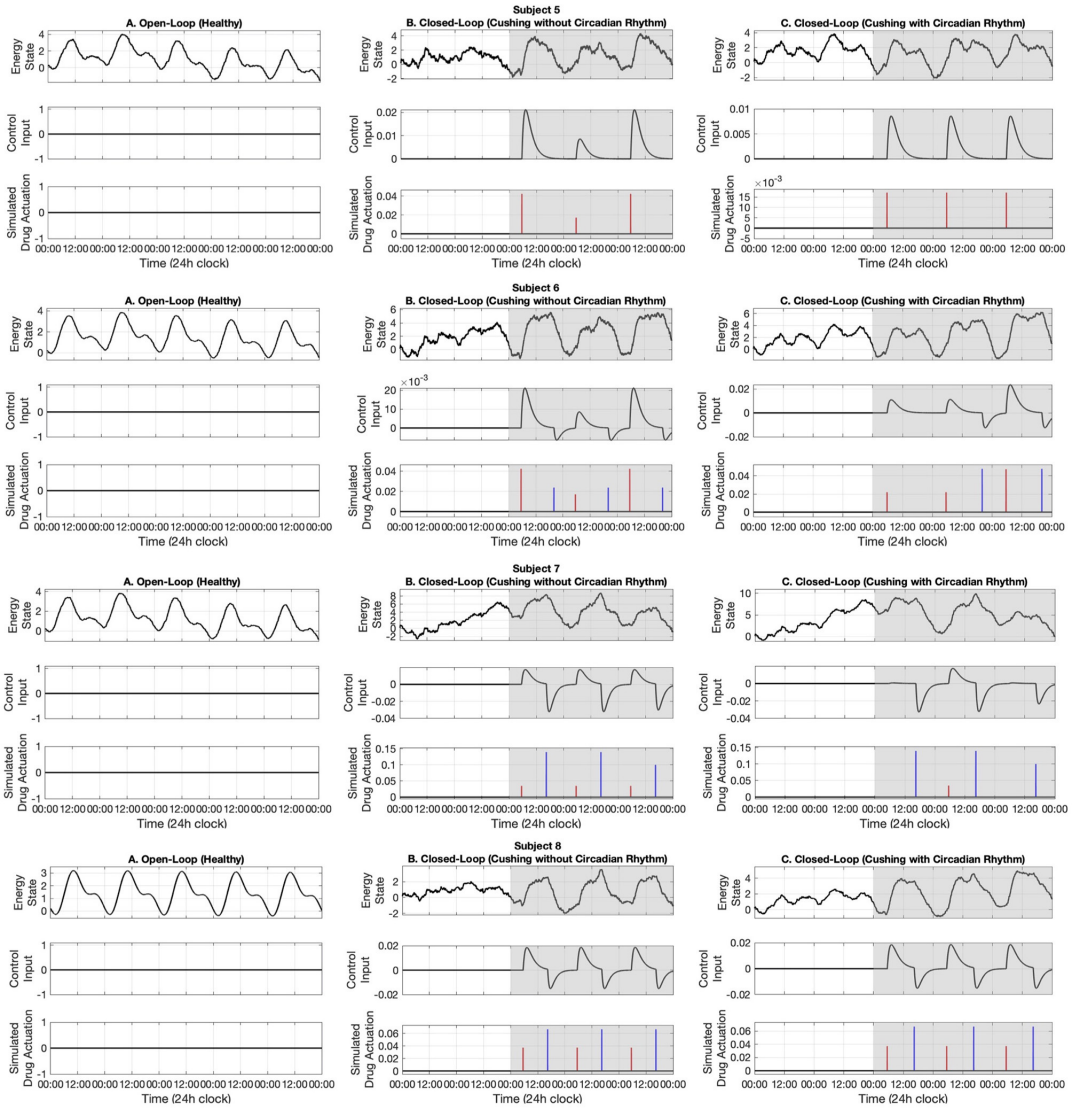
Simulated energy regulation results (Subjects 5–8). For each subject, panel **(A)** displays the open-loop results. Panel **(B)** shows closed-loop results for the Cushing's patients without circadian rhythm, while panel **(C)** shows closed-loop results for the Cushing's patients with circadian rhythm. In each panel: the top sub-panel shows the estimated cognitive energy-related state, the middle sub-panel displays the control input, and the bottom sub-panel depicts the medication injections. Red pulses are related to excitation and the blue pulses are related to inhibition. The white background indicates open-loop simulation (i.e., *u* = 0), while the gray background depicts the closed-loop results.

**Figure 7:**
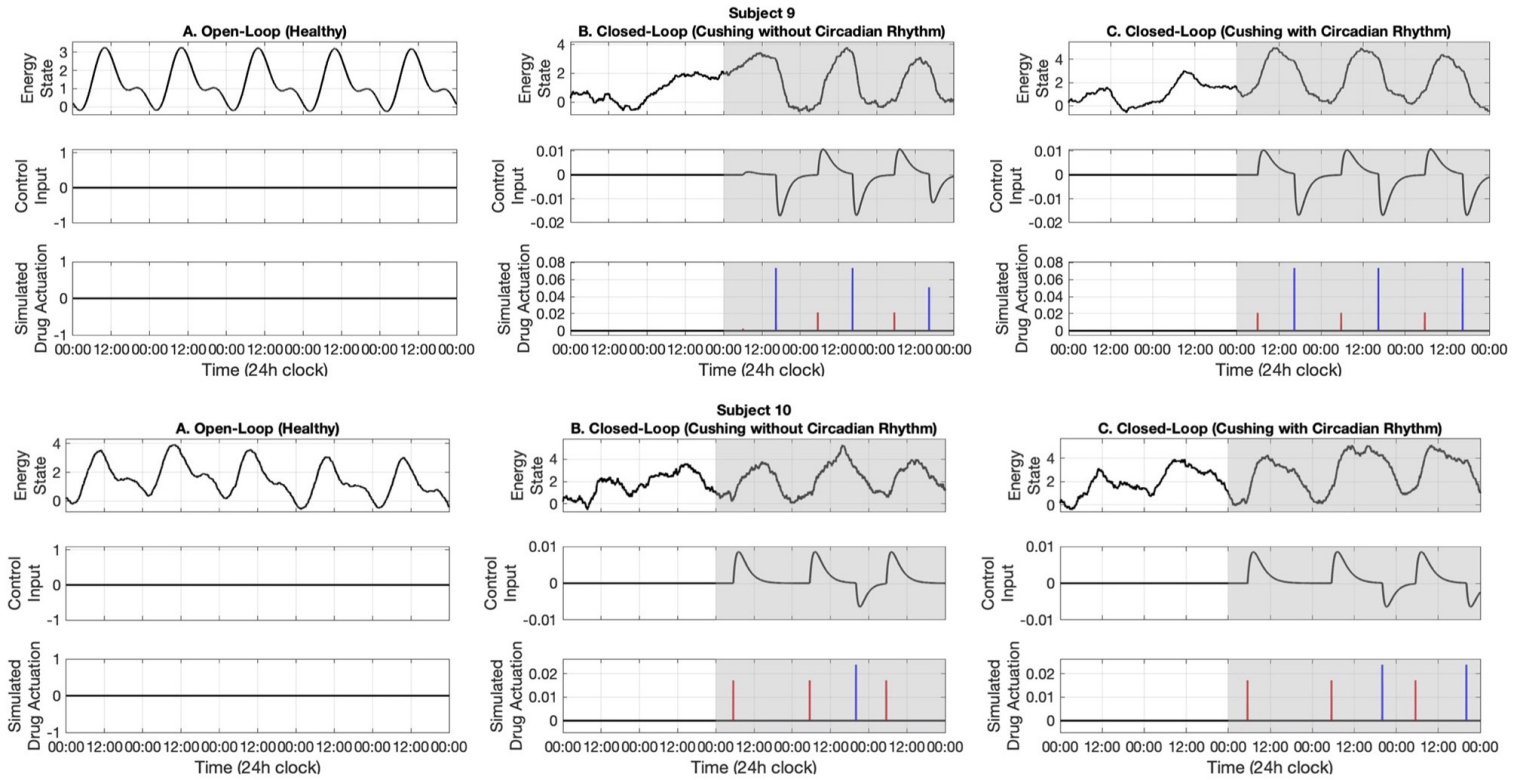
Simulated energy regulation results (Subjects 9 and 10). For each subject, panel **(A)** displays the open-loop results. Panel **(B)** shows closed-loop results for the Cushing's patients without circadian rhythm, while panel **(C)** shows closed-loop results for the Cushing's patients with circadian rhythm. In each panel: the top sub-panel shows the estimated cognitive energy-related state, the middle sub-panel displays the control input, and the bottom sub-panel depicts the medication injections. Red pulses are related to excitation and the blue pulses are related to inhibition. The white background indicates open-loop simulation (i.e., *u* = 0), while the gray background depicts the closed-loop results.

In this section, first we present the open-loop results. Then, we present our real-time closed-loop results for two categories of Cushing's diseases: one without circadian rhythm in their cortisol profiles, and another with circadian rhythm in their cortisol profiles. The results associated with ten simulated subjects are presented in Figures 5–7.

### 3.1. Open-Loop (Healthy Subject)

In the first part, we use data associated with healthy subjects to show the tracking performance. As depicted in the left panels of Figures 5–7, the system tracks the energy state in an open-loop manner. In the middle sub-panel, it is observed that there is not any control in this stage (*u*_*k*_ = 0). Top sub-panels show that the estimated energy state has its peak during the daytime (06:00–16:00) and it drops in the evening. It verifies that we successfully track the energy state in the simulated healthy profiles.

### 3.2. Closed-Loop (Cushing's Patients Without Circadian Rhythm)

In this part, we employ the simulated cortisol data associated with Cushing's patients without circadian rhythm in their cortisol profiles. The results are observed in the middle panels of Figures 5–7. The white and gray backgrounds correspond to the open-loop and the closed-loop periods, respectively. After day 2, the control is activated and the closed-loop system detects an imbalanced energy state (top sub-panel in the middle panels of Figures 5–7). Then, the time and dosage of the required simulated medications are produced by the control system (bottom sub-panel in the middle panels of Figures 5–7). The red pulses stand for the simulated medications with excitation effects, while the blue pulses are associated with the simulated medications with inhibitory effects. Employing the suggested hypothetical medications will lead the generated control input to follow the curves presented in the middle sub-panel of Figures 5–7. Thereafter, starting day 3 of simulation (once the loop gets closed), the energy state is being regulated.

### 3.3. Closed-Loop (Cushing's Patients With Circadian Rhythm)

Similar to the previous case, here we investigate the performance of the proposed closed-loop architecture by making use of simulated Cushing's patients' data together with existing circadian rhythm in their cortisol profiles. The results are presented in the right panels of Figures 5–7. Similarly, the system detects the irregular energy patterns and regulates the energy state variations by designing the corresponding control signals in a closed-loop manner.

## 4. Discussion and Conclusions

Inspired by the fact that CRH plays an undeniable role in internal energy regulation, we proposed our novel approach for regulating the energy-related state using a wearable brain machine interface architecture. In the proposed architecture, we infer one's energy variations by monitoring cortisol data which can be collected using wearable devices in real time (Parlak et al., 2018). We implemented the control algorithm on ten simulated data profiles in healthy subjects and Cushing's patients.

In the offline stage of this research, we simulated the cortisol data for multiple subjects. As it is validated in the literature, we employ stochastic models to simulate multi-day cortisol secretion patterns. Following (Faghih, 2014; Lee et al., 2016; Wickramasuriya and Faghih, 2019b), we consider different gamma distributions for inter-arrival times associated with cortisol secretion impulses. We also assume the pulse amplitudes follow Gaussian distributions. Employing the model parameters that are presented in the manuscript, we simulate cortisol profiles which have day-by-day variability. The stochastic variability existing in model parameters would be viewed as a realistic multi-day case in this *in silico* study. Employing the state-space approach along with EM algorithm, we estimated the model parameters and the forcing circadian function. Using the virtual patient environment, we aimed to track the energy state based on the changes in cortisol secretion times and cortisol upper and lower envelopes (see Figure 2).

With the goal of tracking the energy state in the proposed architecture, we first simulated a real-time open-loop case. In this part, we used the data associated with healthy subjects. In the present study, due to the lack of real-time deconvolution algorithm, we assume the presence or absence of cortisol secretion forms a binary point process and follows a Bernoulli distribution. Besides, we take the cortisol upper and lower bound envelopes as the continuous observations. Utilizing the EM algorithm, we estimated the hidden energy state. As it can be seen in the left panels, with no control implemented (i.e., *u*_*k*_ = 0), the energy state variations in simulated healthy profiles are as desired. It is observed that the energy state is at its peak during the daytime and it drops in the evening. It leads to providing enough energy for daily activity and having well-ordered sleep cycles at evening. In fact, this normal condition is because of the well-balanced cortisol secretion patterns in healthy subjects.

In this research, we assumed that including the hypothetical medications would impact the energy state. Hence, we incorporated the simulated medication dynamics as the actuation while closing the loop. In this regard, we first presented the system identification required to design the control system. To incorporate the corresponding medications in real-world implementation of the proposed closed-loop architectures, it is important to pay appropriate attention to medications' half-lives. In the present design, we assumed that the hypothetical medications have prompt effects on one's energy levels. In the case of utilizing long-acting agents, the rules and membership functions should be revised accordingly. While this step is performed in the offline stage of this research, in the practical case, it is recommended to execute it in real-time to update the medication dynamics according to the subject's response. To design the control, we proposed a knowledge-based fuzzy controller. Employing the estimated energy state, personalized desired levels of energy state, and hypothetical medication dynamics we built the rule base, membership functions, and inference engine (see Figure 2).

Next, we presented the results of the closed-loop system. In this regard, we employed the cortisol data profiles associated with the Cushing's patients. To depict the performance of the closed-loop system, we assumed the control system gets activated starting day 3, which means the system is open-loop (i.e., *u*_*k*_ = 0) in the first 2 days of the simulation. During the open-loop period, we observe that the energy variations do not follow the ideal circadian rhythm. In other words, the patients with hypercortisolism do not have normal cortisol secretion patterns which will cause them to have insufficient energy levels in the day time and experience disturbed sleep cycles at night (Pednekar et al., 2020). Starting day 3, the feedback control system (i.e., *u*_*k*_ ≠ 0) closes the loop (gray background in Figures 5–7). In the closed-loop period, the implemented control system detects undesired energy variations and tries to infer the right time and dosage of the simulated medications in real-time. That is to say, the fuzzy structure receives the estimated energy state, employs the rule base (Table 1) and membership functions (Figure 4), and generates the appropriate control signal. This intermittent control signal is depicted in the bottom sub-panels of Figures 5–7. When low levels of energy are detected, the red pulses would be generated to adjust the dosage of the required medications with excitation effect to provide required energy levels. On the other hand, once undesired high levels of energy are detected in the evening, the medications with the energy lowering effect, i.e., blue pulses, would be suggested to provide the inhibition effect. The required time and dosage of these hypothetical medications are produced by the fuzzy structure. The control actuation signal, which is result of applying these simulated medications, is presented in the middle sub-panels of Figures 5–7. Considering the constraint of using maximum two medications per day (Bouwer et al., 2000), the energy state is regulated in real-time. It is worth mentioning that in the real-world case, the only needed signal for closing the loop is the time and dosage of required medications. Since this simulation study is the first step to show the feasibility of our proposed approach, we simulated hypothetical medication dynamics to include actuation in the virtual patient environment.

In the final part of our results, we presented the outcomes of our proposed structure on simulated cortisol data profiles associated with the Cushing's patients with circadian rhythm in their profiles. While Cushing's patients do not generally have the required circadian rhythm in their cortisol profiles, there exist some patients with some circadian rhythm in their blood cortisol profiles (Lee et al., 2016). This slight circadian rhythm could be assumed to be available in the patients in their early stages of Cushing's disease. Similar to the results of the Cushing's patients without circadian rhythm, our proposed closed-loop architecture successfully detects the energy irregularities and makes the control decisions in real time.

Analyzing the results of multiple subjects, we observe some interesting outcomes. In the results associated with subjects 1, 5, 6, 7, and 10, we see that for some days no blue pulses (i.e., simulated medications with the inhibition effects) are necessary. It might be because energy levels are already low and would not affect their sleep cycles. In these cases, employing only the medications with excitation effects in the morning may lead to energy regulation in the evening too. These results are shreds of evidence of an intrinsic advantage of our proposed closed-loop architectures which is handling the energy regulation in an automated way and suggesting the medications as needed.

**Figure 8:**
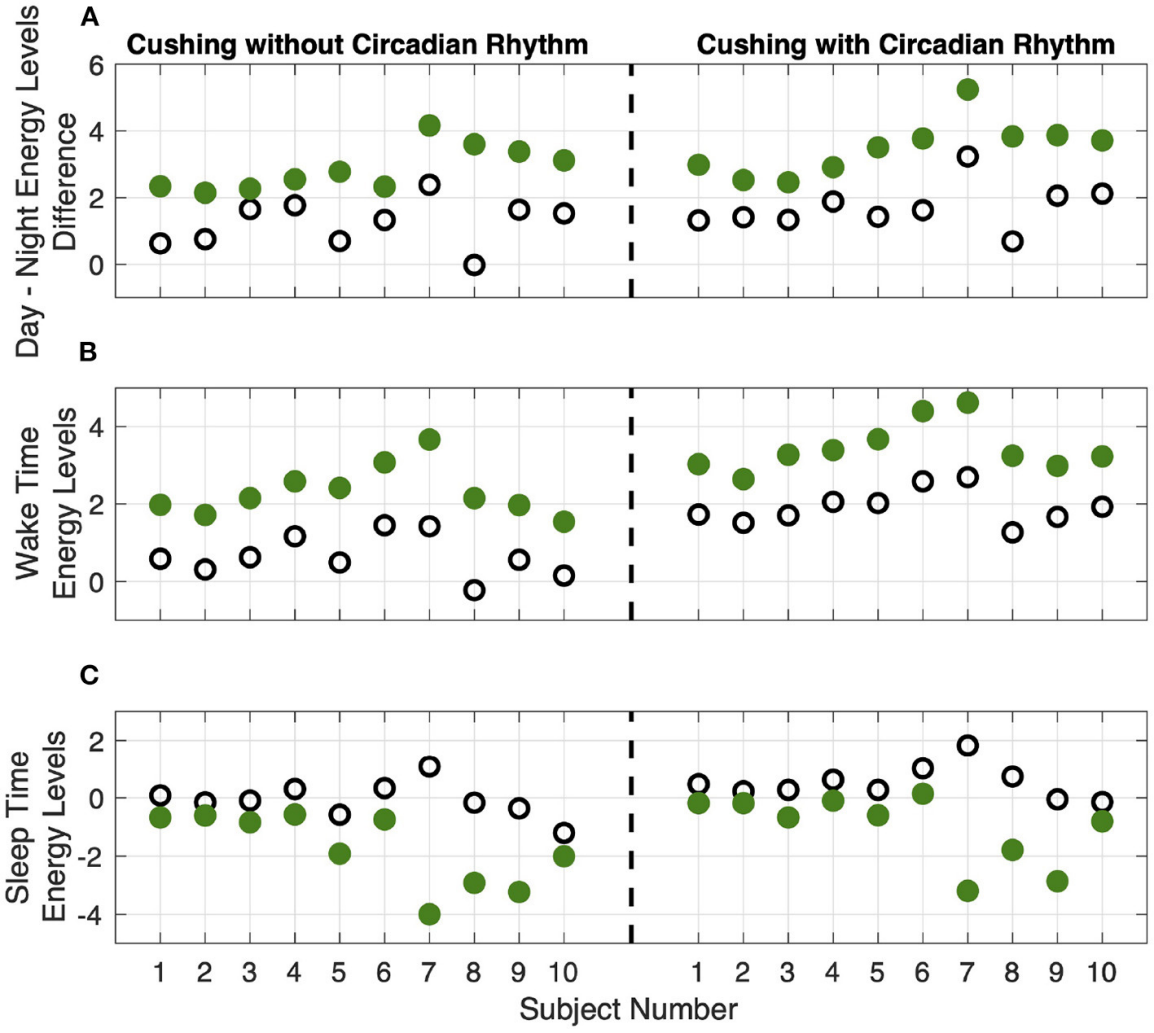
Results analysis. Panel **(A)** shows the difference between average levels of energy in the day and night. Panel **(B)** shows the internal energy growth required for the wake-up time balance. Panel **(C)** shows the decrease in internal energy levels required for sleep time balance. The empty circles and the filled green circles show the results of the open-loop and closed-loop cases, respectively. The left and right sub-panels show the data corresponding to the Cushing patients without and with circadian rhythm in their cortisol profiles, respectively.

**Figure 9:**
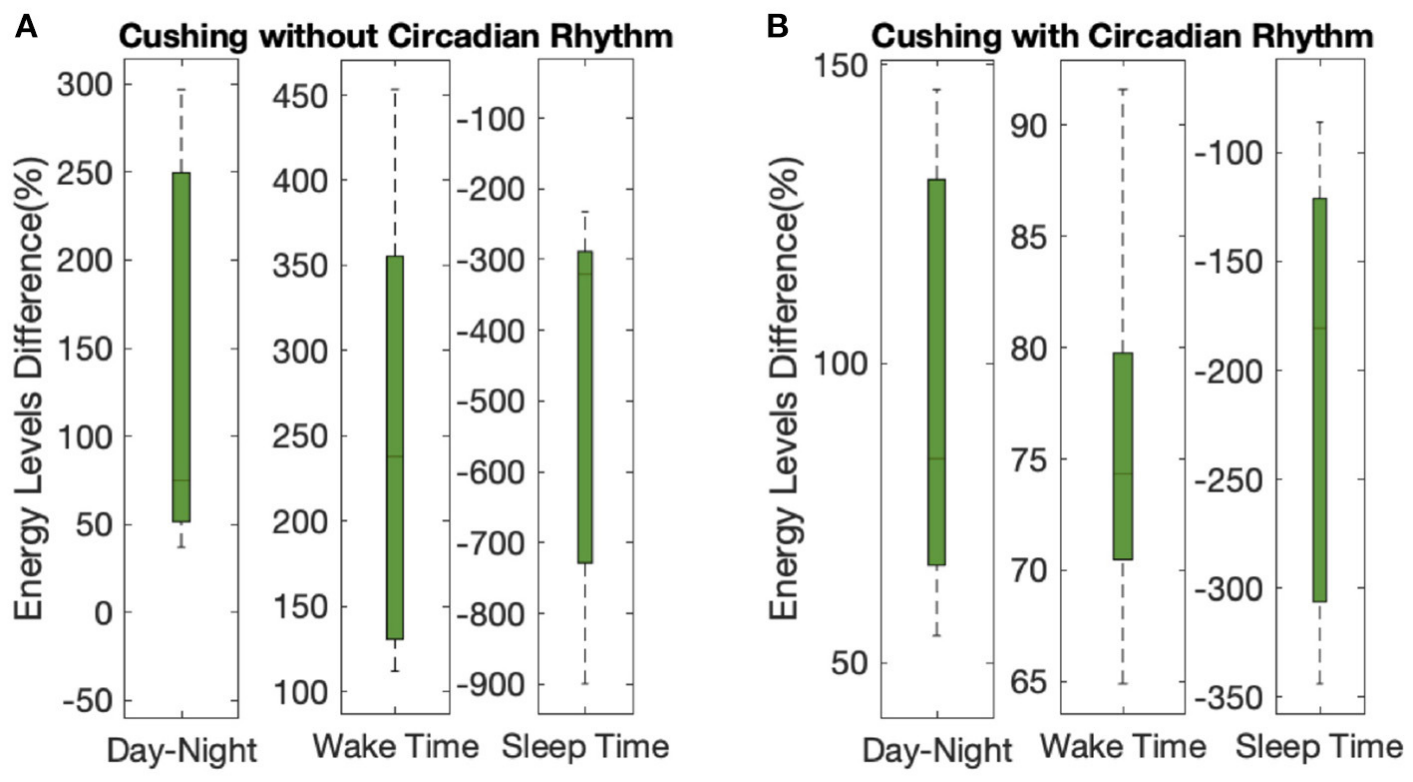
Overall results analysis. The lower- and upper-bounds of the of each box represents the 25th and 75th percentiles of the distribution of each metric for all 10 subjects, and the middle line in each box displays the median. Panels **(A,B)** show the data corresponding to the Cushing patients without and with circadian rhythm in their cortisol profiles, respectively.

To further depict the efficiency of the proposed closed-loop architecture, we define corresponding metrics (Figures 8, 9). As the first criteria, we analyze the effect of closed-loop system in increasing the difference between average levels of energy in the day and night (top panel of Figure 8). As presented, the difference between the average levels of energy in day and night has been increased for all ten simulated subjects in both Cushing's classes (filled green circles compared to the empty circles). As the second criteria, we analyzed the growth of internal energy state in the morning, which will ultimately lead the subjects to wake up with higher levels of energy. To do this task, we compared the growth of energy before the start of the day in both open-loop and closed-loop cases (middle panel of Figure 8). The observed growth of energy in all simulated subjects will help them to wake up with having more energy required for their daily activities. As the final criteria, we compared the drop of energy levels late at evening (bottom panel of Figure 8). It demonstrates how the proposed closed-loop architecture resulted in decreasing the energy levels required for a better sleep cycle. As presented in the bottom panel of Figure 8, the internal energy state in patients with Cushing's disease are not decreased sufficiently in the evenings (empty circles). However, in the closed-loop case, by applying the required medications, the simulated energy state has been dropped more efficiently which will further help the subjects to experience more balanced sleep cycle at night.

Analyzing the results on all the simulated subjects, the difference between the average energy levels in day and night in Cushing's patients without and with circadian rhythm in their cortisol profiles is improved by 140% and 97%, respectively (left sub-panels in Figure 9). The growth in the energy levels before the wake time in both classes of Cushing's patients is improved by 245% and 75%, respectively (middle sub-panels in Figure 9). Similarly, the average drop in the energy levels required for sleep time regulation is improved by 473% and 208% in simulated Cushing's patients without and with circadian rhythm in their profiles has been, respectively (right sub-panels in Figure 9). This analysis verify how our proposed architecture is effective in regulating energy levels in a virtual patient environment.

In the offline stage of this research, we simulated multi-day data profiles for healthy subjects and subjects with Cushing's disease. It is worth mentioning that this stage of simulating multi-day data profiles is because of the lack of technology for real-time cortisol measurements. Future advances in wearable technologies would provide the opportunity to continuously monitor the cortisol data and design a system that could take care of inter- and intra-subjects fluctuations. In the present study, we assumed that the suggested medications could be successful in lowering or increasing energy levels. In practical implementation, there exist multiple factors that might cause the proposed architectures (i.e., using suggested medications to regulate internal energy state) to fail and result in less efficiency:

- Diverse sensitivity to glucocorticoid hormones among individuals might prevent to observe similar energy adjustments in response to the medications (Inda et al., 2017).
- Sever dysregulation of the HPA axis, which happens in some endogenous Cushing's syndrome cases, could only be treated by removing the pituitary or adrenal tumor(s) (Nieman et al., 2015).

Implementing the proposed wearable brain machine interface architecture on multiple simulated cortisol profiles, we demonstrated that we can reach energy regulation in hypercortisolism. Simulated results verify that the proposed closed-loop approach has great potential to be utilized in real life. In the prospective practical system, a real-time deconvolution algorithm should be utilized to derive the CRH secretion times. Employing the proposed approach, in addition to taking advantage of wearable devices, which may monitor blood cortisol levels in real-time, the time and dosage of the required medications would be regulated in a closed-loop automated manner. Since the cortisol variations are influenced by a variety of physiological and psychological factors, a future direction of this research could be including additional information from multiple sources while designing the closed-loop system. In the prospective architectures, a multi-input multi-output system will take the information from multiple sources and make the required decisions about taking the medications (i.e., dosage and time). It results in an increase in medications' efficiency and minimize their possible side effects. Future directions of this research could be incorporating all possible medications and designing the control algorithms with the capability to choose among them. Another possible future direction could be including the system identification process for each medication inside the real-time system. As a result, the way that each specific person responds to a particular medication will be monitored to update the medication dynamics in real-time. Consequently, the personalized control design would be more efficient.

## Data Availability Statement

The original contributions presented in the study are included in the article/Supplementary Material, further inquiries can be directed to the corresponding author/s.

## Author Contributions

RF conceived and designed the study. RF and HF developed the algorithms and analysis tools. HF performed the research, analyzed the data, and wrote the manuscript. HF, J-OH, and RF revised the manuscript. All authors contributed to the article and approved the submitted version.

## Conflict of Interest

RF is a co-inventor on a provisional patent that designs decoders for estimating energy based on cortisol observations. The remaining authors declare that the research was conducted in the absence of any commercial or financial relationships that could be construed as a potential conflict of interest.

## Publisher's Note

All claims expressed in this article are solely those of the authors and do not necessarily represent those of their affiliated organizations, or those of the publisher, the editors and the reviewers. Any product that may be evaluated in this article, or claim that may be made by its manufacturer, is not guaranteed or endorsed by the publisher.

## Supplementary Material

Supplementary Figure 1 Simulated multi-day cortisol profiles (Subjects 1–4). For each subject, panel **(A)** displays the healthy profile, panel **(B)** shows the profiles associated with the Cushing's patients without circadian rhythm, and panel **(C)** depicts the profiles associated with the Cushing's patients with circadian rhythm. Each panel displays cortisol levels (black curve), upper bound envelops (orange curve), and lower bound envelopes (green curve).

Supplementary Figure 2 Simulated multi-day cortisol profiles (Subjects 5–8). For each subject, panel **(A)** displays the healthy profile, panel **(B)** shows the profiles associated with the Cushing's patients without circadian rhythm, and panel **(C)** depicts the profiles associated with the Cushing's patients with circadian rhythm. Each panel displays cortisol levels (black curve), upper bound envelops (orange curve), and lower bound envelopes (green curve).

Supplementary Figure 3 Simulated multi-day cortisol profiles (Subjects 9 and 10). For each subject, panel **(A)** displays the healthy profile, panel **(B)** shows the profiles associated with the Cushing's patients without circadian rhythm, and panel **(C)** depicts the profiles associated with the Cushing's patients with circadian rhythm. Each panel displays cortisol levels (black curve), upper bound envelops (orange curve), and lower bound envelopes (green curve).

Supplementary Table 1 Infusion and clearance rates associated with the ten simulated cortisol profiles.

Supplementary Table 2 Parameters used to generate forcing function. $\left(I_{k}=\sum_{i=1}^{2}\alpha_{i}\sin\left(\frac{2\pi ik}{1440}\right)+\beta_{i}\cos\left(\frac{2\pi ik}{1440}\right)\right)$.
