# Supplementary figures and images for: Closed-Loop Fuzzy Energy Regulation in Patients With Hypercortisolism via Inhibitory and Excitatory Intermittent Actuation
Source: Front Neurosci. 2021 Aug 9;15:695975. doi: 10.3389/fnins.2021.695975 (PMC8381152; doi:10.3389/fnins.2021.695975)

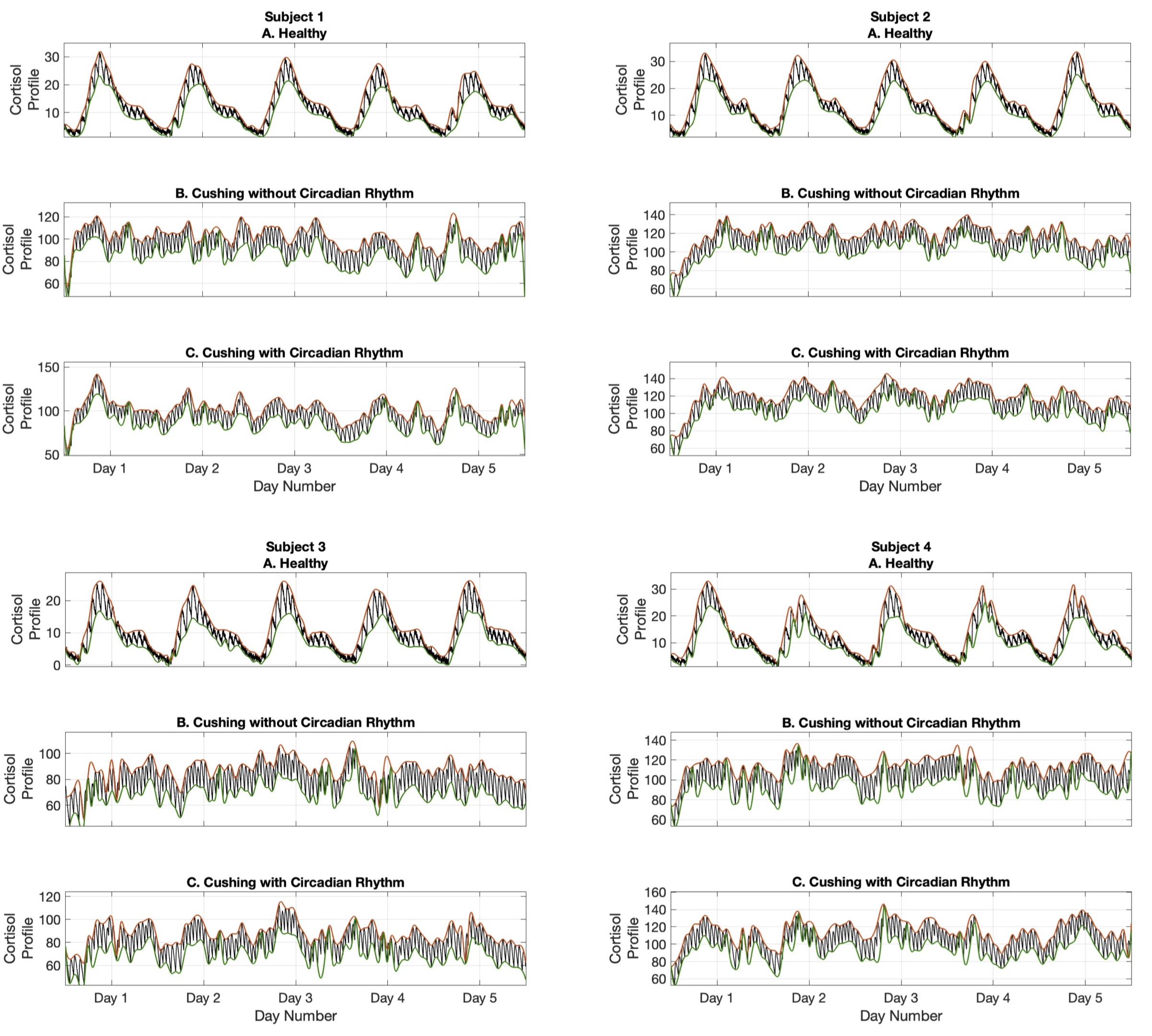

Supplement: Supplementary Figure 1 — Simulated multi-day cortisol profiles (Subjects 1–4). For each subject, panel (A) displays the healthy profile, panel (B) shows the profiles associated with the Cushing's patients without circadian rhythm, and panel (C) depicts the profiles associated with the Cushing's patients with circadian rhythm. Each panel displays cortisol levels (black curve), upper bound envelops (orange curve), and lower bound envelopes (green curve). [file Image_1.JPEG]

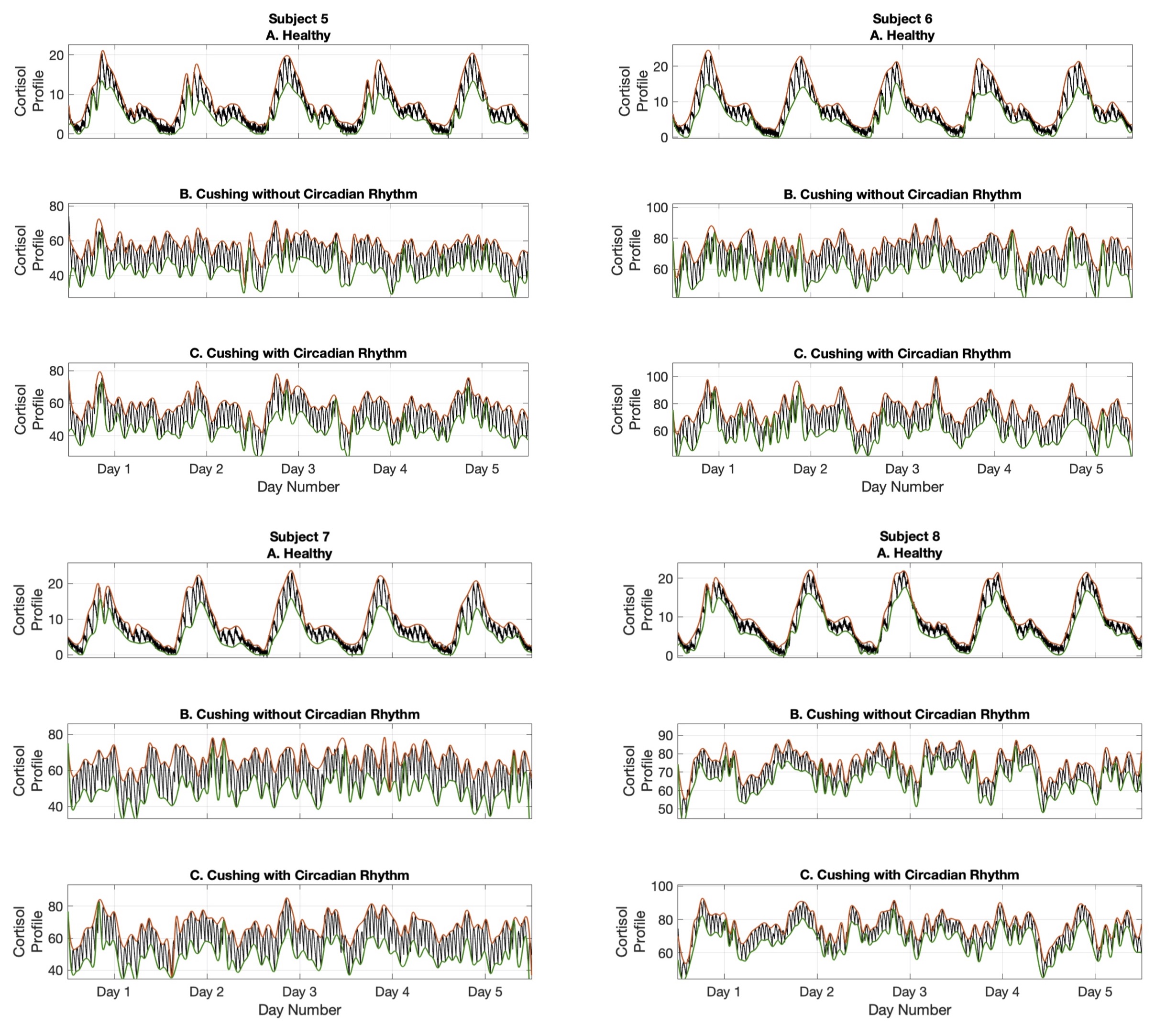

Supplement: Supplementary Figure 2 — Simulated multi-day cortisol profiles (Subjects 5–8). For each subject, panel (A) displays the healthy profile, panel (B) shows the profiles associated with the Cushing's patients without circadian rhythm, and panel (C) depicts the profiles associated with the Cushing's patients with circadian rhythm. Each panel displays cortisol levels (black curve), upper bound envelops (orange curve), and lower bound envelopes (green curve). [file Image_2.JPEG]

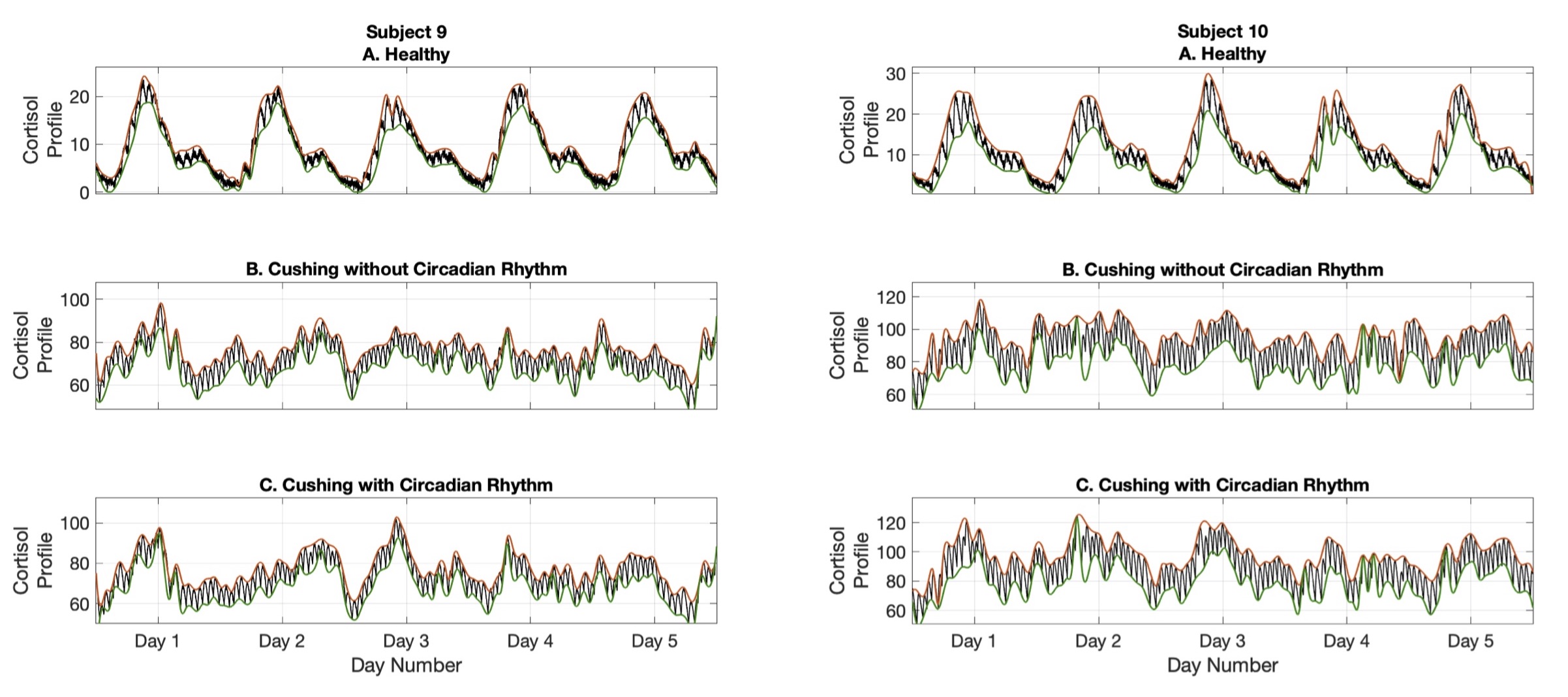

Supplement: Supplementary Figure 3 — Simulated multi-day cortisol profiles (Subjects 9 and 10). For each subject, panel (A) displays the healthy profile, panel (B) shows the profiles associated with the Cushing's patients without circadian rhythm, and panel (C) depicts the profiles associated with the Cushing's patients with circadian rhythm. Each panel displays cortisol levels (black curve), upper bound envelops (orange curve), and lower bound envelopes (green curve). [file Image_3.JPEG]
