# Supplementary material for: Closed-Loop Fuzzy Energy Regulation in Patients With Hypercortisolism via Inhibitory and Excitatory Intermittent Actuation
Source: Front Neurosci. 2021 Aug 9;15:695975. doi: 10.3389/fnins.2021.695975 (PMC8381152; doi:10.3389/fnins.2021.695975)
Supplement: Supplementary Table 1 — Infusion and clearance rates associated with the ten simulated cortisol profiles. [file Table_1.PDF]

Table S1. Infusion and clearance rates associated with the ten simulated cortisol profiles.

Subject number	$\zeta_1(min^{-1})$	$\zeta_2(min^{-1})$
1	0.0739	0.0067
2	0.0762	0.0057
3	0.0921	0.0082
4	0.1248	0.0061
5	0.0585	0.0122
6	0.0726	0.0095
7	0.0799	0.0107
8	0.0365	0.0091
9	0.0361	0.0090
10	0.0864	0.0073

Table S2. Parameters used to generate forcing function ($I_k = \sum_{i=1}^{2} \alpha_i \sin(\frac{2\pi ik}{1440}) + \beta_i \cos(\frac{2\pi ik}{1440})$).

Subject number	Case	α_1	β_1	α_2	β_2
1	A	0.00565	0.00108	−0.00046	−0.00585
	B	0.00104	0.000003	0.00370	−0.00186
	C	0.00280	0.00129	0.00286	−0.000546
2	A	0.00577	0.00100	−0.00062	−0.00533
	B	0.00048	−0.00100	0.000011	−0.000006
	C	0.00300	−0.00035	−0.00017	−0.00376
3	A	0.00538	−0.000013	−0.00087	−0.00657
	B	−0.000002	0.000002	0.000009	−0.000002
	C	0.00267	−0.00007	−0.00073	−0.00530
4	A	0.00566	0.00101	−0.00051	−0.00626
	B	0.00125	0.000006	0.000001	−0.00236
	C	0.00285	0.00203	0.00085	−0.00595
5	A	0.00522	0.00191	−0.000014	−0.00693
	B	0.00284	0.00069	−0.000006	−0.00099
	C	0.00393	0.00080	0.00334	−0.00659
6	A	0.00534	0.00190	0.00105	−0.00672
	B	0.00163	−0.00233	−0.00064	−0.00453
	C	−0.00295	−0.00095	0.00238	−0.00911
7	A	0.00516	0.00220	0.00017	−0.00659
	B	−0.00079	−0.00235	−0.00031	−0.00381
	C	0.00241	−0.00082	0.00245	−0.00895
8	A	0.00560	0.00003	−0.00064	−0.00613
	B	0.00023	0.00063	0.00190	0.00060
	C	0.00186	−0.000032	−0.00001	−0.00613
9	A	0.00543	0.00114	−0.00117	−0.00637
	B	0.00104	0.00188	−0.00152	0.00095
	C	0.00267	0.00286	−0.00140	−0.00306
10	A	0.00559	0.00124	−0.00049	−0.00632
	B	0.00283	−0.00052	0.00002	0.00288
	C	0.00445	−0.00003	−0.00054	−0.00416
